# Deciphering transcriptome patterns in porcine mesenchymal stem cells promoting phenotypic maintenance and differentiation by key driver genes

**DOI:** 10.3389/fcell.2024.1478757

**Published:** 2024-11-06

**Authors:** Nadia Khaveh, René Buschow, Julia Metzger

**Affiliations:** ^1^ Institute of Animal Genomics, University of Veterinary Medicine Hannover, Hannover, Germany; ^2^ Research Group Veterinary Functional Genomics, Max Planck Institute for Molecular Genetics, Berlin, Germany; ^3^ Microscopy and Cryo-Electron Microscopy Facility, Max Planck Institute for Molecular Genetics, Berlin, Germany

**Keywords:** mesenchymal stem cells, bone marrow, transcriptome, RNA-seq, pig, Mini-Lewe, differentiation

## Abstract

Mesenchymal stem cells (MSC) are fibroblast-like non-hematopoietic cells with self-renewal and differentiation capacity, and thereby great potential in regeneration and wound healing. MSC populations are heterogeneous not only inherently, but also among different model species. In particular, porcine MSC serve as a frequently used resource for translational research, due to pigs’ distinctive closeness to human anatomy and physiology. However, information on gene expression profiles from porcine MSC and its dynamics during differentiation is sparse, especially with regard to cell surface and inner cell markers. In this study, we investigated the transcriptome of bone marrow-derived MSC and its differentiated cell types in a minipig breed for experimental research, known as Mini-LEWE, using bulk mRNA sequencing. Our data highlighted Rap1 signaling and downstream pathways PI3K-Akt and MAPK signaling as potential players for the maintenance of stemness of BM-MSC. In addition, we were able to link the process of differentiation to changes in the regulation of actin cytoskeleton. A total of 18 “BM-MSC differentiation driver markers” were identified, potentially promoting the process of differentiation into adipocytes, chondrocytes as well as osteocytes. Our results offer a new perspective on the molecular phenotype of porcine BM-MSC and the transcriptional responses in new differentiated progeny.

## 1 Introduction

Mesenchymal stem cells (MSC) are adult stem cells with characteristic proliferative and differentiation capacities (Friedenstein et al., 1970; Tavassoli and Crosby, 1968; Caplan, 1991). Research goes back to a long history of studies on the multipotency and mesengenesis of these mesenchymal stromal cells and their potential use for wound healing, immunomodulation and regenerative medicine (Caplan, 1994; Caplan, 1995; Horwitz et al., 2005). One of the common sources of MSC is non-hematopoietic cell population of the bone marrow, contributing to tissue homeostasis, immune regulation and tissue repair along with immune cells (Le Blanc and Ringden, 2007; Kfoury and Scadden, 2015; Sangiorgi and Panepucci, 2016).

MSC populations are known to be inherently heterogeneous and show different phenotypic and behavioural subtypes (Mets and Verdonk, 1981). In addition, subtle yet significant diversity is found in MSC from different tissue- and species sources, sampling procedures and culture conditions (Costa et al., 2021; Dominici et al., 2006). However, there are several MSC-specific characteristics common in all cultured MSC populations, namely their adherence to plastic surface, the ability to multilineage differentiate to adipocytes, osteocytes, chondrocytes *in vitro* as well as the expression of specific surface antigens (Dominici et al., 2006; Choudhery et al., 2022). In human, bone-marrow derived MSC (BM-MSC) were found to present the surface “cluster of differentiation” (CD) markers CD29, CD44, CD73, CD90 and CD105 in more than 95% of the cell population, and CD14 (CD11b), CD34, CD45, CD19 (or CD79α), and HLA-DR in less than 2% of cell population (Dominici et al., 2006; Choudhery et al., 2022). However, in animal models, the composition of cell surface markers is slightly different: For example, cultured BM-MSC from horses were shown to express CD29, CD44, CD90, CD105, CD166 and but lack CD34, CD45 and CD79α expression (Bundgaard et al., 2018). Similarly, in bovine BM-MSC, transcriptomic profiles suggested CD29, CD44 and CD73 to be highly expressed, whereas CD90 as one of the strongest MSC-indicators, held no and/or lower expression in comparison to human and horse MSC (Kato et al., 2004; Danev et al., 2024). It was demonstrated that bovine BM-MSCs share more common functionally relevant gene expression profiles with human BM-MSCs than compared to murine BM-MSCs and thus highlighted the particular potential of non-murine cells for translational studies (Danev et al., 2024). With regard to the surface marker CD105 in bovine BM-MSC, its role was controversially discussed as it was either found to be not highly expressed or missing (Kato et al., 2004; Danev et al., 2024). Similar findings were made for porcine BM-MSC, which were proposed to strongly express CD29, CD90, and CD44, but were found to have a lower CD105 expression compared to human BM-MSC, and no CD45 expression (Juhásova et al., 2011; Prinz, 2017). In contrast, in another study, the complete absence of CD73 and CD105 expression in porcine BM-MSC was highlighted and underlined the need for further investigations on the characteristics of these cells (Schweizer et al., 2020). Despite these studies reporting on selected markers, a full list of potential marker genes remains elusive. For successful detection, it was proposed that high throughput sequencing of RNA helps to improve stem cell characterization, which is otherwise limited due to the absence of appropriate antibodies against markers for various selected species (Dawson and Lunney, 2018). Subsequently, RNA sequencing was successfully applied for defining cell-specific mRNA expression to interrogate the spectrum of cell surface proteins, known as the surfaceome (Pais et al., 2019)

Pigs represent a particular valuable non-primate model for translational and clinical medicine to target disease, cell therapy, immunomodulation, regeneration and xenotransplantation, due to its similarities to human anatomy and physiology, and its relatively short as well as seasonal-independent gestation time (Lee et al., 2007; Krupa et al., 2007; Walters and Prather, 2013; Hatsushika et al., 2014; Khatri et al., 2015; Kawamura et al., 2015; Ock et al., 2016; Tseng et al., 2018; Fisher, 2021). Therefore, it is important to gain a comprehensible knowledge of the molecular phenotype of porcine MSC. So far, the majority of studies on the characterization of MSC relied on flow cytometry, Real-Time PCR (RT-qPCR) and microarrays. In addition, RNA sequencing approaches are used in human and few model species to not only characterize MSC, in particular BM-MSC, but also to unravel the underlying differentiation mechanisms and hierarchies (Danev et al., 2024; Roson-Burgo et al., 2014; Cho et al., 2017; Haga et al., 2024; Kanazawa et al., 2021; Zhan et al., 2019; Koch et al., 2022).

In pigs, RNA sequencing of MSC from subcutaneous adipose tissue and synovial joints was performed for different commercial large breeds in order to understand molecular mechanisms related to mesengenic formation and paracrine signaling in general, as well as to study diseases such as metabolic syndrome (Li et al., 2023; Ponsuksili et al., 2024; Conley et al., 2018; Eirin et al., 2017; Pawar et al., 2020). In a microarray analysis of MSC from Yorkshire crossbreed pigs, the transcriptome of adipose-derived and bone-marrow derived cells was compared and studied for its *in vitro* osteogenic and adipogenic differentiation (Monaco et al., 2012). It was highlighted that BM-MSC had larger angiogenic, osteogenic, migration and neurogenic capacities, presumably more suitable for specific therapeutic applications (Monaco et al., 2012). Moreover, expression profiling of porcine BM-MSC was done to study cryopreservation and treatment with histone deacetylase inhibitors to identify cellular responses related to cell stress, development and differentiation (Gurgul et al., 2017; Gurgul et al., 2018). These studies emphasized that BM-MSC represent the gold standard for its use in tissue regeneration and thereby require thorough molecular phenotyping and investigation of its differentiation processes (Monaco et al., 2012; Monaco et al., 2009).

In this research paper, our goal is to study the transcriptome profiles of BM-MSC and its differentiated cell lineages specifically in the miniature pig breed Mini-LEWE, using bulk mRNA sequencing. We aim to investigate the transcriptional expression of known or potential new candidate stem cell surface markers as well as potential intracellular markers in BM-MSC and its derivatives.

## 2 Material and methods

### 2.1 Sample collection

In this study, we obtained BM-MSC of the iliac crest of three 80-day-old Mini-LEWE piglets. The piglets underwent euthanasia in a two-step process using intramuscular injection of Azaperone (2 mg/kg) and Ketamine (20 mg/kg) and subsequent intracardial application of T61 (Tetracaine hydrochloride, Mebezonium iodine and Embutramide cocktail (6mL/50kg, MSD Tiergesundheit - Intervet Deutschland GmbH, Germany). This procedure was approved by the animal welfare officer of the University of Veterinary Medicine Hannover (“Tötungsanzeige”, ID TIHO-T-2020-9), in accordance with national and international guidelines. After euthanasia, the laterofrontal of the ilium bone was exposed and a biopsy needle (Jamshidi with T-handle, Lehnecke, Germany) was used to aspirate bone marrow from the iliac crest. The aspirate was transferred to an EDTA-coated collection tube (BD vacutainer, New Jersey, United States), suspended in ice-cold CO_2_-independent medium (Gibco, New York, United States) with 2% Glutamax (Gibco), and transported to cell culture laboratory. In addition, Peripheral Blood Mononuclear Cells (PBMC), representing differentiated hematopoietic cells from bone-marrow, were used as the control samples to the non-hematopoietic undifferentiated BM-MSC. They were obtained from full progeny of the sampled pigs and proceeded into RNA isolation.

### 2.2 Cell isolation and culture

The acquired bone marrow samples were treated with Red Blood Cell Lysis Buffer (Roche, Basel, Switzerland) to remove the erythrocytes from the cell suspensions. Next, the samples were transferred to cell culture flask T-175 (Sarstedt, Nuembrecht, Germany) and incubated in DMEM (Gibco) with 10% Foetal Bovine Serum at 5% CO_2_ and 37°C for 24 h. Then, the media were changed and cells were maintained initially in MesenPRO RS Medium (Gibco) and all further passages in Mesenchymal Stem Cell Growth Medium 2 (MSC-GM2, PromoCell, Heidelberg, Germany). Cells at passages four (P4) and five (P5) were used for differentiation and RNA isolation.

### 2.3 Directed differentiation of mesenchymal stem cells

BM-MSC-multipotency was tested by directed adipogenesis, chondrogenesis and osteogenesis during two consecutive passages P4 and P5. For this purpose, BM-MSCs were detached from their vessels with TrypLE Express Enzyme (1X) (Gibco), stained with Trypan Blue Solution, 0.4% (Gibco) and counted on Neubauer chamber (Roth, Karlsruhe, Germany). Next, 6 × 10^5^ viable cells were seeded into three 10 cm^2^ petri dishes (2,00,000/dish), one dish for each type of differentiation. Cells were maintained for 24 h at 5% CO_2_ and 37°C in MSC-GM2 allowing them to recover and attach. Adipogenesis was induced using StemPro Adipogenesis Differentiation Kit (Gibco) for 10 days (media exchanged every second day). After differentiation, cells were maintained for another 7 days on Human Adipocyte Maintenance Media (Cell Application, San Diego, United States). Chondrogenesis was promoted using StemPro Chondrogenesis Differentiation Kit (Gibco) for 14 days and media was refreshed every second day. The differentiated chondrocytes were maintained for another 7 days on Chondrocyte Growth Medium (PromoCell). Osteogenesis was induced using Mesenchymal Stem Cell Osteogenic Differentiation Medium (PromoCell) for 24 days and the cells maintained for another 7 days in Minimum Essential Medium α, nucleosides (Gibco) containing 10% FBS and 2% Glutamax.

### 2.4 Staining and microscopy

After differentiation, cells from each cell type were transferred to µ-Slide 8 Well (ibidi GmbH, Graefelfing, Germany), maintained for 2 days, fixed with 4% ice-cold formaldehyde for exact 10 min at room temperature, and washed three times with distilled water. Transmitted light images of adipo-, chondro- and osteocytes were captured using the Celldiscoverer 7 (Zeiss, Oberkochen, Germany). Due to the lack of a condenser in the platform, the so-called phase gradient contrast (PGC) was utilized. The PGC images were automatically acquired with a self-adjusted aperture, so the cellular fine structures could be scanned throughout multiwall formats without edge or meniscus artefacts.

Next, the water was removed from the wells and each cell type was specifically stained. Fixed adipocytes were washed once with 60% isopropanol for 5 min, covered with filtered working Oil Red O solution (3 times stock Oil Red O (3 mg/ml) in 2 times distilled water) for 5 min at room temperature, and finally washed with distilled water until all excessive stain was removed. Fixed chondrocytes were washed once with PBS, then covered with 1% Alician blue in 0.1N HCl staining solution for 30 min at room temperature. Upon removal of the staining solution, the excess stain washed away with 0.1N HCl. Furthermore, fixed osteocytes were stained with 2% Alizarin Red solution (pH 4.2) for 3 min at room temperature, and washed with distilled water. All stained cells were maintained in 250 μL distilled water. RBG images were acquired using Axio Observer Z1 (Zeiss) equipped with RGB (red, green, and blue) camera.

### 2.5 RNA isolation and library preparation

In total, all three BM-MSC samples from three Mini-Lewe, six adipocyte, chondrocyte and osteocyte samples each underwent RNA isolation and library preparation (Supplementary Table S1). In addition, the RNA of three PBMC samples was isolated.

All cells were scraped from the plastic surface and resuspended in TRIzol, then transferred into innuSPEED Lysis Tubes X (Innuscreen GmbH, Berlin, Germany) for homogenization on a pre-cooled SpeedMill PLUS (Analytik Jena GmbH, Jena, Germany) for two interval steps. Subsequently, RNA isolation was performed based on TRIzol user guide provided by Invitrogen (Massachusetts, United States). The quality and integrity of the isolated RNA was controlled using a High Sensitivity RNA ScreenTape assay on 4200 TapeStation system (Agilent, Santa Clara, United States). Samples were selected for library preparation based on RNA integrity numbers (RIN) of >8 in BM-MSCs as well as >6 in PBMCs (due to a higher RNA-fragmentation rate of PBMCs in general).

### 2.6 RNA sequencing and data processing

RNA libraries of 24 samples (three BM-MSCs, six adipocytes, six chondrocytes, six osteocytes and three PBMCs) were prepared using NEBNext Ultra II Directional RNA Library Prep Kit for Illumina (NEB, Ipswich, United States) and Unique Dual Index Primer Pairs of NEBNext Multiplex Oligos for Illumina kit (NEB). Libraries were set in equal molarity and sequenced for 70 million reads 2 × 100 bp on an Illumina NextSeq200. The obtained data were quality controlled, pre-processed and mapped based on *Sus scrofa* 11.1 genome reference, as previously described (Khaveh et al., 2023).

### 2.7 Differential gene expression analysis

The raw counts of the mapped reads were extracted using the STAR *quatMode* (version 2.7.9a, (Dobin et al., 2013) and were analysed using DESeq2 in R environment (version 1.44.0, (Love et al., 2014). Prior to differential expression analysis (DEA), preanalytical data quality control based on regularized logarithm (*rlog*) transformation and principal component analysis (PCA) was performed as recommended by Love et al. (2014). In addition, the Euclidian distance of all samples were calculated and clustered to observe their similarity and correlation. DEA was run to identify the unique transcriptome profile of Mini-Lewe BM-MSCs in contrast to PBMCs (control samples) and secondly to observe the differential transcriptomes of BM-MSCs and their differentiated cell types. Thus, six samples from each differentiated cell types (adipocytes, chondrocytes, and osteocytes) were contrasted separately with expression profile of BM-MSC as control. After the DEA for each aforementioned set, the genes with the absolute log2 fold change (|log2FC|)>2 and false discovery rate (padj) < 0.05 were considered as significantly differentially expressed. Finally, we identified and extracted gene expression patterns of cell surface markers and their differential expression information in our porcine BM-MSC and its differentiated cell lineages by comparing our dataset to a compiled list of all known CD markers (Engel et al., 2015) as well as notable MSC markers in human (Miller-Rhodes, 2023; Uder et al., 2018).

### 2.8 Enrichment analysis

First, the lists of differentially expressed genes (DEG) of each contrast, namely of BM-MSCs, adipocytes, chondrocytes and osteocytes, were overlapped with human orthologues acquired from Ensembl Biomart and submitted to enrichR tool (R package, version 3.2, (Chen et al., 2013; Kuleshov et al., 2016; Xie et al., 2021; Jawaid, 2023)) for enrichment with four gene-set databases “KEGG_Human_2021,” “GO Biological Process 2023,” “Reactome_2022” and “Jensen TISSUES.” All term lists were filtered for *p-*value < 0.05. The notable pathway terms highlighted in “KEGG_Human_2021” were further investigated manually on KyotoEncyclopedia of Genes and Genomes website (KEGG: https://www.genome.jp/kegg/pathway.html) for pig (*sus scrofa*) specified pathways.

### 2.9 Differential exon usage

To observe the frequency of exon usage and therefore predict post-transcriptional changes BM-MSC and its derivative cell types, DEXSeq tool (version 1.50.0 (Anders et al., 2012; Reyes et al., 2013)) was used in R environment. First, the annotations of the pig reference genome from Ensembl Biomart were transformed into a TxD object using “makeTxDbFromBiomart” from GenomicFeatures package (Lawrence et al., 2013) and collapsed into counting bins. The counting bins were used to count the number of overlapping exons and read fragments from the aligned reads (Aligned.out.bam files by STAR) using “*summarizeOverlaps*” from GenomicAlignments package (Lawrence et al., 2013). At this stage, the data was split into two objects, one holding MSCs and PBMCs data and another containing MSCs and its derivative cell types. Next, the counted overlapped exons were fitted into a generalized linear model (GLM) with the formula “*∼sample + exon + cell_type*:*exon*” normalized based on size factors estimation (“*estimateSizeFactors*”) and dispersed (“*estimateDispersions*”). The interaction of condition (cell type) and exon (from aforementioned GLM) are compared on Chi squared distribution to establish a *p-*value. Finally, the data was tested for differential exon usage and exon fold changes were estimated based on samples’ cell type. The result was summarised and filtered for significance threshold of |log2FC| > 2 and padj < 0.05.

### 2.10 Weighted gene co-expression network analysis (WGCNA)

We investigated the association of the expressed genes and their correlations with the cell type by modulating a hierarchical clustering and constructing gene networks with a high probability of co-expressing using the R package Weighted Gene Co-expression Network Analysis (WGCNA) (version 1.72-5. (Langfelder and Horvath, 2008; Langfelder and Horvath, 2012). For this purpose, the normalized and stabilized data matrix from DESeq2 analysis was used to build a topological overlapping matrix with soft-thresholding power value of three. The next step of network construction was performed following our previous suggestions (Khaveh et al., 2023). Furthermore, the correlations between each module and cell type were test using Fisher test. Genes from the significantly correlated modules were functionally enriched as described above for functional enrichment of DEGs.

### 2.11 Fluorescence staining and microscopy

For validation of the expression of cell surface markers and functional elements using fluorescent microscopy, we seeded 1,000 cells each cell lineage in dark-walled flat-bottomed 96 wells (Greiner Bio-One GmbH, Frickenhausen, Germany) and fixed as described above in Section 2.4. Next, the fixed cells were rinsed in PBS and permeabilized for 1 h blocked using Normal Donkey Serum Block (NDSB: 1% w/v BSA, 2% v/v Normal Donkey serum, 0.1% v/v Triton X, 0.05 v/v % Tween-20) for 30 min. Primary antibodies against CD105 (mouse anti-pig, Abcam Cat#ab53318, Cambridge, United Kingdom), CD29 (Mouse anti-pig, Cat#561496, BD Pharmingen, New Jersey, United States), CD90 (Mouse anti-pig, Cat#561972, BD Pharmingen) were diluted in 1:500 in the identical NDSB solution and incubated at 4°C overnight. Further primary antibody C7 (mouse anti-human, Proteintech Cat# 66908-1, Illinoise, United States) were diluted in 1:1,000 in NDSB, applied to the wells and incubated for 1 h at room temperature. The antibody solutions were washed out of the well by flushing the well with three times 1 × PBS. Secondary Antibodies as well as phalloidin (1 μg/mL) and Hoechst (0.5 μg/mL) were also diluted in the NDSB and incubated together in one well for 1 h at room temperature before a final flushing with three times PBS.

For evaluation of cellular protein contents (CD marker, or any endogenous content) we used Zeiss Celldiscoverer 7 running under Zen Blue 3.5. All experiment consisted several 1,000s of individual position per condition in large mosaics. The acquisitions were carried out fully automated using a surface detection strategy for stabilizing the focus position under controlled temperature. The acquisitions were carried out with a 20x, NA0.7 or 20x, NA0.95 objectives and a 1x or 2x post magnification the use camera chip was a 12 megapixel Axiocam 712. In Combination with Abbes resolution limit we had a typical lateral (XY) pixel size of 0.352 or 0.258 µm/pixel. The acquired images underwent a standard hierarchical image analysis strategy. Briefly nuclei were detected based on their fluorescence intensity, with fixed intensity thresholds, close by objects were separated by water shedding. The resulting masks were filtered towards an area in between 75 and 800 μm^2^ and a circularity in between 0.6 and 1 (dimensionless). From this primary objects our routine automatically dilated 5 pixel before a secondary with a width around the nuclei of 100 pixel were drawn. Within this region the marker signals were quantified. Our fluorescence data is shown as total fluorescence intensity signal. Typically, we analyzed in between ∼2,000 and 20,000 single cells per condition in more than 30 dimension. Finally, the obtained data for C7 and actin were analyzed using “ggbetweenstat” package (Patil, 2021) with parametric pairwise Welch’s t-test and false discovery rate (FDR) < 0.05.

## 3 Results

### 3.1 Morphology of the undifferentiated and differentiated BM-MSC

**FIGURE 1:**
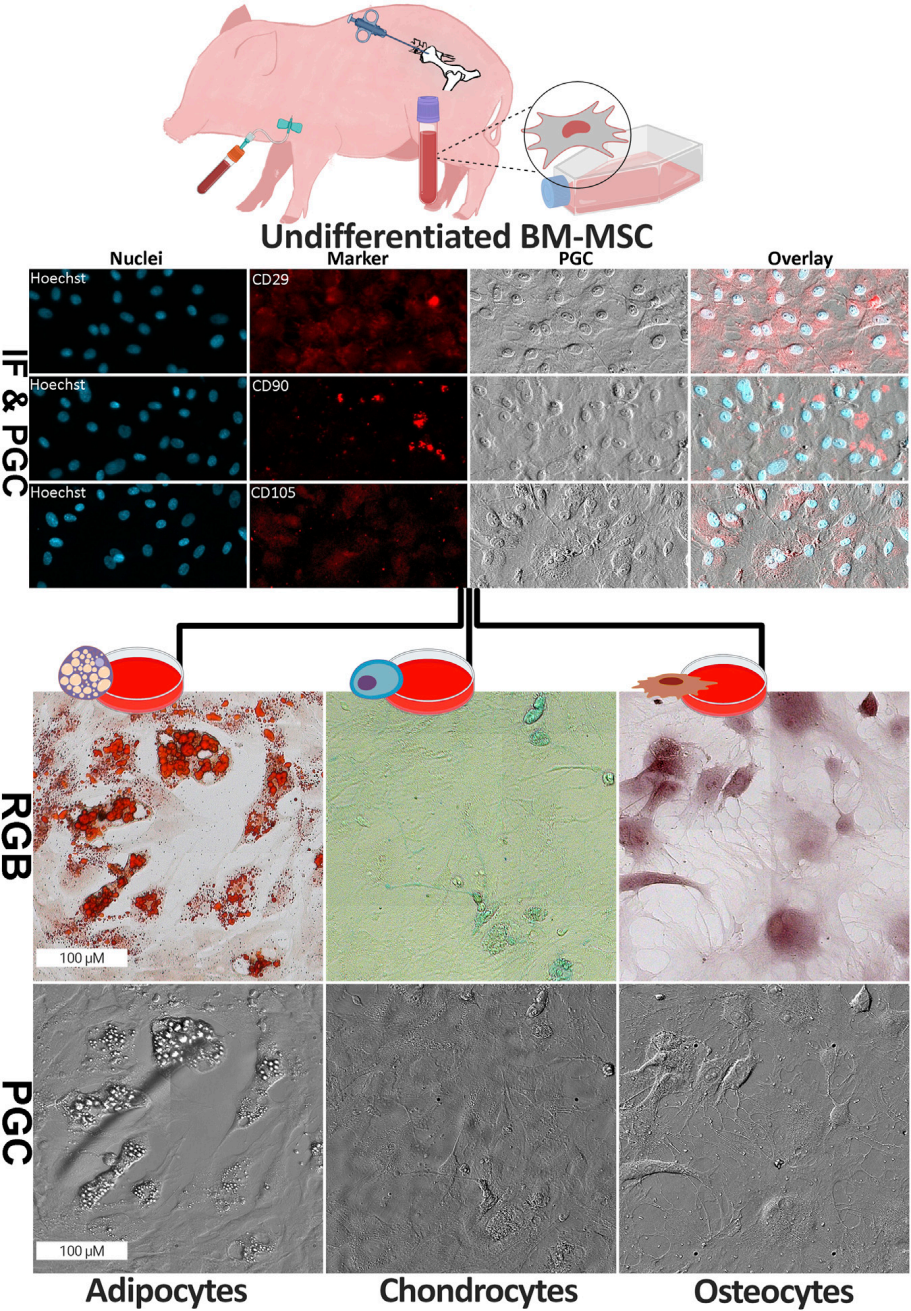
Morphology of undifferentiated and differentiated BM-MSC. (top) The formalin-fixed undifferentiated BM-MSC were investigated using phase gradient contrast (PGC) technique as well as immunofluorescence imaging (IF) for surface markers with anti-CD29, anti-CD90, anti-CD105 and counterstain (Hoechst). PGC capture of the cytoskeleton structure of these cells was done without any staining. (bottom) Successful differentiation of BM-MSC into three cell lineages, namely adipocyte, chondrocyte and osteocyte, was confirmed using cell type specific staining (Oil Red-O for adipocytes, Alician blue for chondrocytes and Alizarin Red for osteocytes; RGB = red, green and blue). In addition, changes in cell morphology were highlighted using PGC technique (Image partially created by BioRender, agreement number: VN275VWCHA).

First of all, we investigated the characteristics of the BM-MSC population using three cell surface markers, CD29, CD90 as well as CD105. The image analysis showed that all three CD markers were expressed strongly in the BM-MSC (Figure 1). Next, we aimed to validate the capacities of our approach to differentiate into osteo-, chondro- and adipocytes. Therefore, we used the well-established above described classical histological staining protocols on our differentiated cells and captured images with a contrast transmitted light strategy as well as RGB images of the identical cells in a second microscope. The undifferentiated BM-MSC were observed in small colonies and well as spread out as single cells. The cells in dense colonies had a spindle-like morphology, while the cells surrounding the colonies in less populated areas displayed a spread-out cytoplasm with a visible cytoskeleton organisation. In the adipocytes, Oil Red O stain highlighted lipid vacuoles distinctly in bright red (Figure 1). The chondrocytes’ phenotype was observed with 1% Alician blue as the glycosaminoglycan became visible with fine blue signals within and surrounding the cells. In addition, osteocyte differentiation was confirmed by highlighting the calcium content of these cells stained in bright red by 2% Alizarin red stain.

### 3.2 Transcriptome profiles of undifferentiated BM-MSC

**FIGURE 2:**
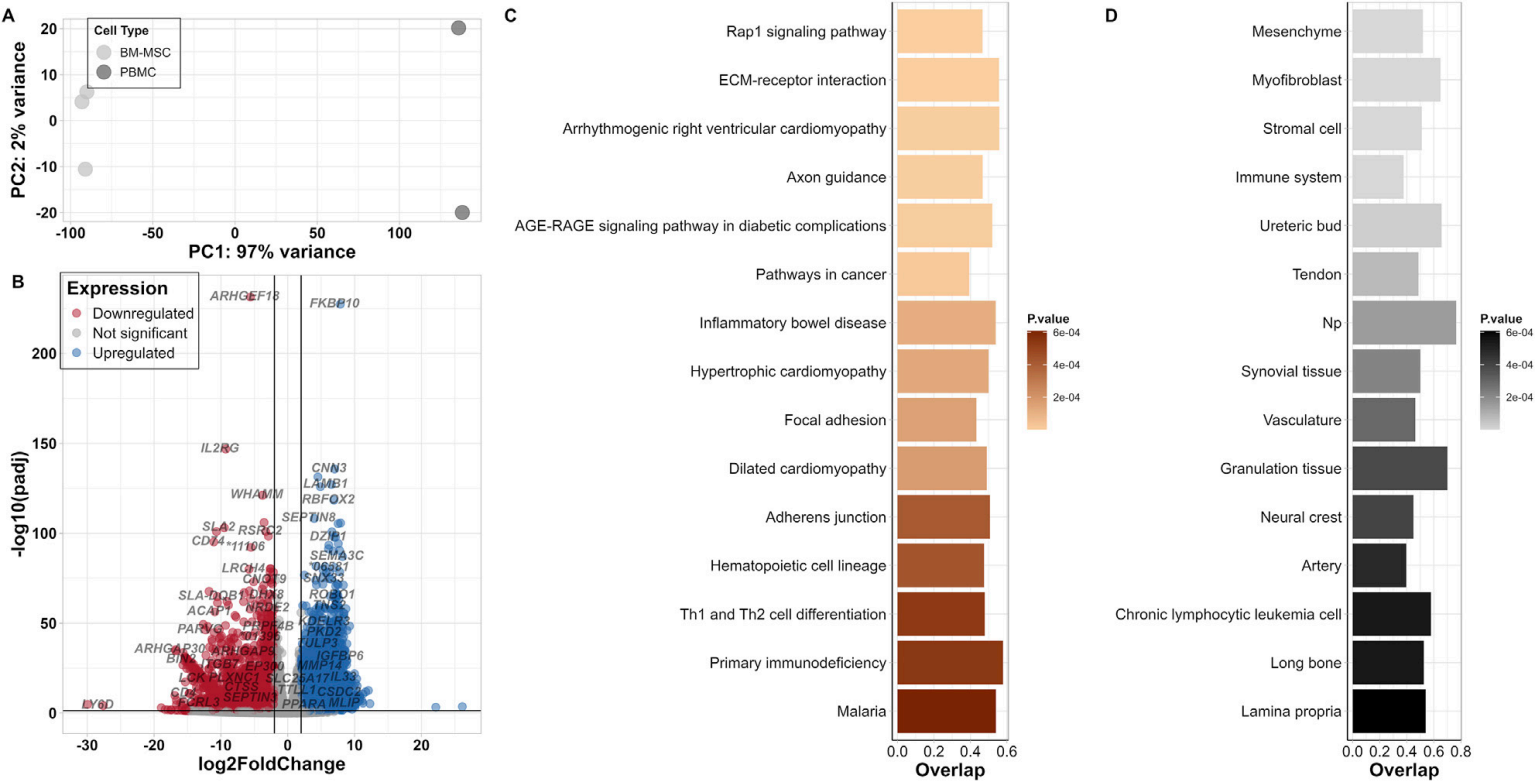
Differentially expressed genes in BM-MSC in contrast to PBMC. **(A)** PCA plot for BM-MSC and PBMC samples **(B)** Volcano plot of DEGs; log2FC is on the *x*-axis and padj on the *y*-axis (* = ENSSSCG000000). Thresholds of significance are shown on the left and right of the intersection lines. **(C)** Top 15 significant KEGG pathways (based on KEGG_Human_2021 database) and **(D)** Jensen TISSUEs displayed for their number of overlapped genes and *p*-value.

Cell type-specific expression profiles provide essential knowledge for cell identification and marker-based characterisation of cells *in vitro*. In our investigation of BM-MSC, we called 14,019 out of 35,670 annotated genes to be expressed based on normalised counts per million (cpm), of which 91.4% were protein-coding and 7.3% were long non-coding RNAs (lncRNA). Furthermore, the Euclidian distance test for BM-MSC showed a high dissimilarity to PBMC (Supplementary Figure S1). Each cell type displayed a clustering within its replicates. In comparison to PBMCs, BM-MSCs revealed a distinct separation on the first principal component (PC1) by 97% variance between the two groups, whereas the variance among the individual samples within each group (PC2) was less than 1% in PCA (Figure 2A). Within the transcriptome of porcine BM-MSC, we could identify 253 expressed genes out of 371 CD markers known in human.

In our DEA, we identified 6,285 DEGs within the significance threshold padj <0.05 and |log2FC| >2, of which 2,994 genes were upregulated and 3,291 were downregulated (Figure 2B; Supplementary Table S2). Furthermore, we could identify 146 DEGs from the list of human CD markers.

Enrichment analysis of DEGs for “KEGG_Human_2021” pathway revealed significantly enriched pathways such as Rap1 signalling pathway and ECM-receptor interactions (Figure 2C; Supplementary Table S3). In addition, “Jensen TISSUES” database highlighted the involvement of tissues and cells within the bone marrow referring to BM-MSCs in contrast to PBMCs such as “Mesenchyme,” “Stromal cell,” “Immune system” and “Chronic lymphocytic leukemia cell” (Figure 2D; Supplementary Table S3). From other two databases, “GO Biological Process 2023” and “Reactome_2022” enriched terms such as “Extracellular matrix organization (GO:0030198, R-HAS-1474244),” “Collagen fibril organization (GO:0030199),” “Collagen formation (R-HAS-1474290),” “External encapsulating structure organization (GO:0045229),” “Embryonic skeletal system development (GO:0048706)” and also immunomodulatory processes such as “Inflammatory Response (GO:0006954),” “B Cell Receptor Signaling Pathway (GO:0050853),” “Regulation Of T Cell Activation (GO:0050863)” were highlighted. In addition, gene families such as *COL*, *ADAM* and *HOX* genes were frequently observed in these clusters (Supplementary Table S3).

### 3.3 Mesengenic differentiation shifts transcriptome of BM-MSC

One of the widely known properties of BM-MSCs is the multipotency and the ability to differentiate into certain cell lineages, namely adipocyte, chondrocyte, and osteocyte (Caplan, 1991). In order to identify the transcriptome changes of BM-MSC after differentiation into new cell-phenotypes, we compared the different expression profiles. PCA revealed a 32% variance (PC1) among all four cell types, whereas on the PC2 dimension, the distinct separation among adipocytes, osteocytes and BM-MSC can be observed (Supplementary Figure S2).

**FIGURE 3:**
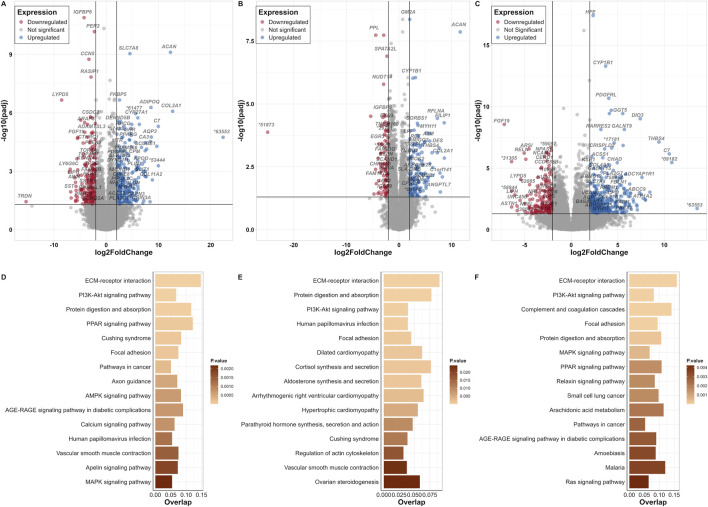
BM-MSC and its derivative cell-lineages. Volcano plots of DEGs from contrasts of **(A)** adipocyte versus BM-MSC (control), **(B)** chondrocyte versus BM-MSC (control), and **(C)** osteocyte versus BM-MSC (control) datasets; log2FC on the *x*-axis and padj on the *y*-axis (* = ENSSSCG000000). Thresholds of significance are shown on the left and right of the intersection lines. Top 15 significant KEGG pathways (based on KEGG_Human_2021 database) displayed for their number of overlapped genes and their *p*-value score for **(D)** adipocyte versus BM-MSC (control), **(E)** chondrocyte versus BM-MSC (control), and **(F)** osteocyte versus BM-MSC (control) datasets.

About fifteen thousand genes were identified in adipocytes, chondrocytes and osteocytes, respectively. These included 88% protein coding genes and 10% lncRNAs in all three cell types. We identified 483 DEGs in adipocytes compared to BM-MSC, of which 259 were upregulated and 224 downregulated (Figure 3A; Supplementary Table S4). In chondrocytes, 246 DEGs (149 up- and 97 downregulated) were identified (Figure 3B; Supplementary Table S5). Furthermore, osteocytes revealed 598 DEGs (387 up- and 211 downregulated) (Figure 3C; Supplementary Table S6).

Enrichment analysis based on “KEGG_Human_2021” pathway revealed significant common terms in all three DEG datasets such as “ECM-receptor interaction,” “PI3K-Akt signalling pathway2” and “Focal adhesion” (Figures 3D–F). From other databases, terms such as such as “Mesenchyme” and “Abdominal adipose tissue,” “Bone matrix” and “Adipocyte,” “Fat cell differentiation (GO:0045444),” “Positive regulation of cell differentiation (GO:0045597)” and “Fatty acid transport (GO:0015908)” for adipocytes, “Long bone,” “Epiphyseal growth plate” and“ Chondrocyte cell line,” “Skeletal system development (GO:0001501) for chondrocytes as well as “Long bone,” “Mesenchyme,” “Bone matrix,” “Tibia,” and “Osteoblast cell line” for osteocytes were highly significant (Supplementary Tables S7–S9).

**FIGURE 4:**
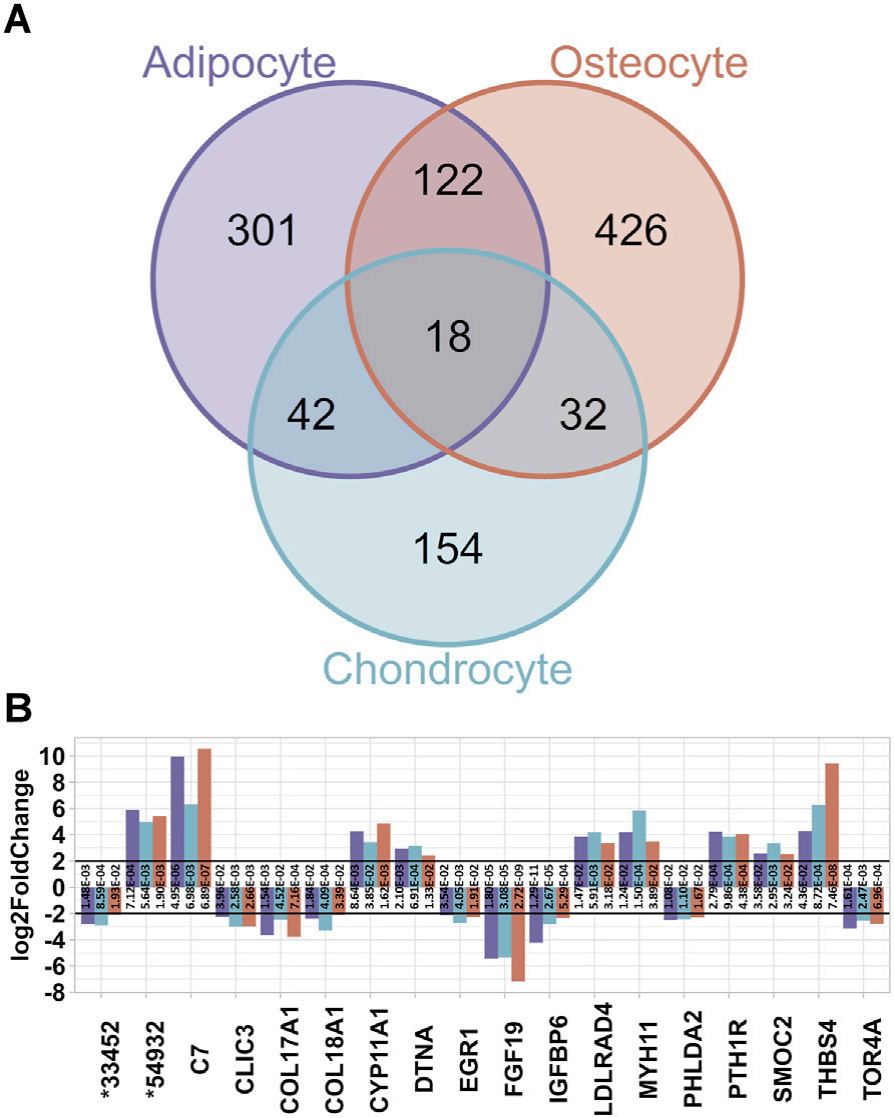
Common DEGs among the three differentiated lineages. **(A)** Venn diagram for the number of DEGs from each dataset. In total, 18 DEG are common. **(B)** Overview of the common 18 DEGs and their log2FC from all three datasets. The padj values of each test is displayed on each bar (* = ENSSSCG000000).

**FIGURE 5:**
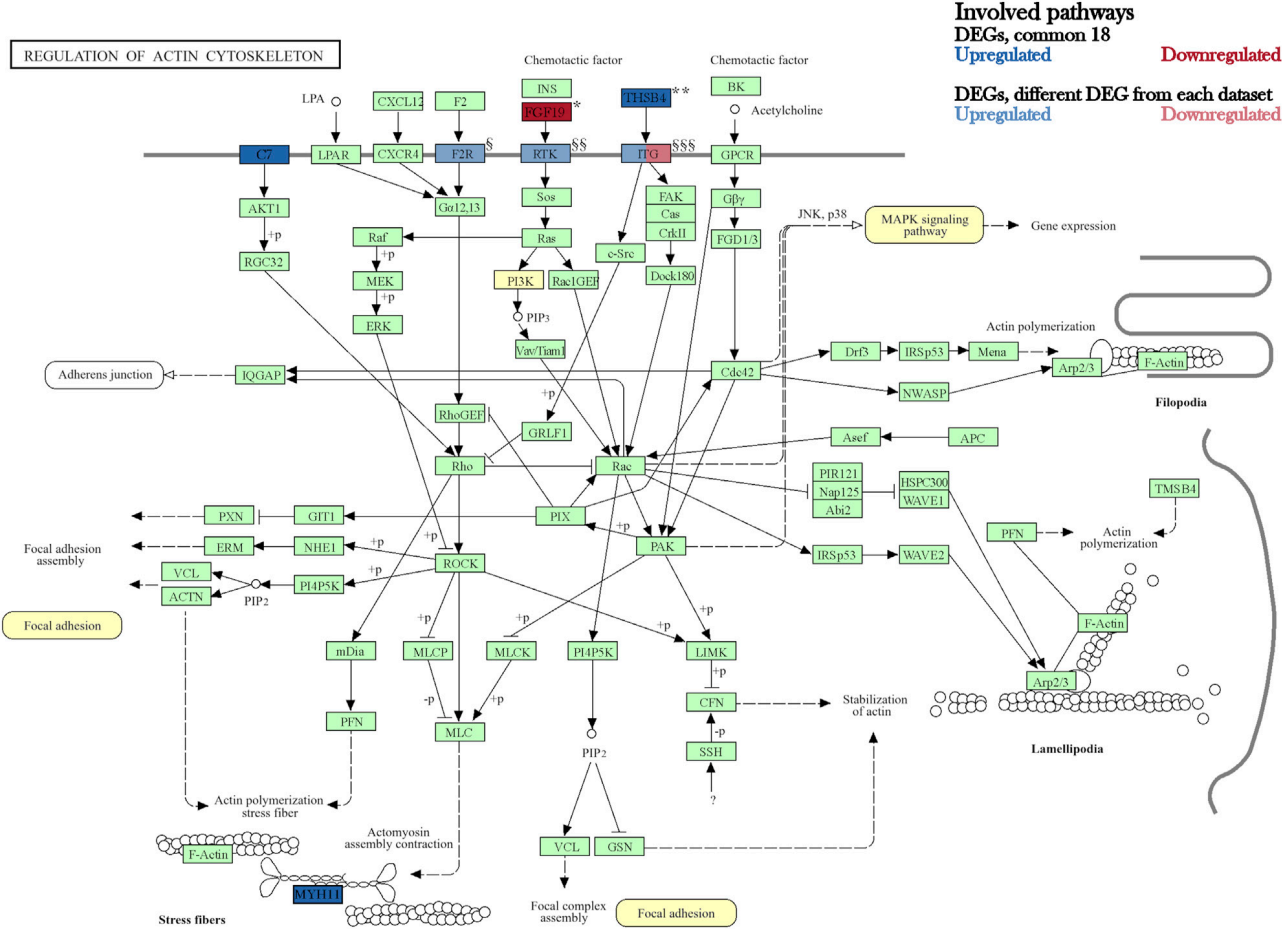
KEGG pathway “Regulation of actin cytoskeleton” (ssc04810). Modified figure according to KEGG database. The highlighted genes are DEGs observed in all three datasets. Individual gene names from gene clusters “Focal adhesion, ssc04510” and “PI3K/Akt signaling pathway, ssc04151” were added to the figure below (Copyright permission 240859 by Kanehisa labs).

Furthermore, comparisons of the three DEG lists of adipocytes, chondrocytes and osteocytes contrasted to BM-MSC revealed 301 DEGs unique for adipocytes, 426 for osteocytes and 154 for chondrocytes (Figure 4A). Additionally, 32 DEGs were detected both for chondrocytes and osteocytes, whereas 42 DEGs were called for adipocytes and chondrocytes as well, and 122 DEGs in adipocytes and osteocytes, respectively. Subsequently, 18 DEGs were common among all three datasets. These 18 DEGs showed similar pattern of up- or downregulation among all three DEG lists (Figure 4B). Interestingly, ten of these DEGs were also differentially expressed in BM-MSC compared to PBMCs. In contrast, genes such as *C7*, *MYH11*, *EGR1*, *CLIC3*, and *THBS4* were unique for the differentiated cell lineages. Among these, *C7*, *MYH11*, *THSB4* and *FGF19* were assigned to a common pathway “Regulation of actin cytoskeleton” in KEGG database (KEGG path ID: ssc04810). According to KEGG, this pathway was not only highlighted to influence PI3K-Akt and MAPK pathway, which contained several other DEGs (e.g. *FGFR2*, *PDGFRA*, and several *ITGA* genes) but was also shown to be affected by the upstream paths of “Focal adhesion signalling” (KEGG path ID: ssc04510, Figure 5).

**FIGURE 6:**
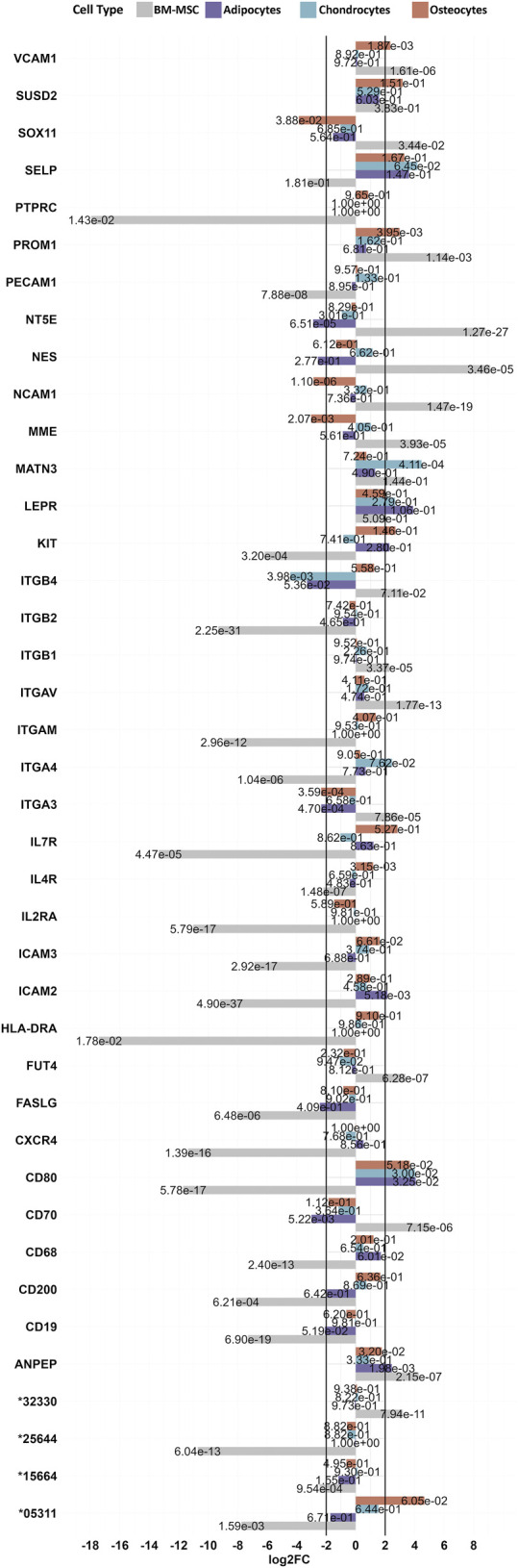
List of MSC markers with significant changes in porcine BM-MSC. The bar chart represents 40 significantly differentially expressed known cell surface markers in BM-MSC (vs. PBMC). Expression levels are compared to DEGs of adipocytes, chondrocytes and osteocytes (all vs. BM-MSC). Padj values for each dataset are presented on its related bars.

Finally, 67 notable MSC marker genes according to our list of all known CD markers as well as notable MSC markers in human (Miller-Rhodes, 2023; Uder et al., 2018), which are not restricted to BM-MSC only, were investigated for the gene expression among our identified DEGs for their log2FCs of BM-MSC relative to PBMC as well as BM-MSC relative to adipocytes, chondrocytes or osteocytes (Figure 6). Among these markers, 21 of the analogous genes, such as *PTPRC* (CD45), *HLA-DRA* (MHC-II), *CD200* and *CD19* were downregulated in BM-MSC (in comparison with PBMCs). In contrast, 14 DEGs, including *ITGB1* (*CD29*), *Thy-1* (CD90, pig annotation: ENSSSCG00000032330), *FUT4* (CD15), *VCAM1* (CD106), *MME* (CD10), *CD70*, *NCAM1* (CD56), *NT5E* (CD73) were upregulated in BM-MSC (in comparison with PBMC). Additionally, 26 genes coding for MSC markers did not show significant differential gene expression in BM-MSC, however some markers such as *ENG* (CD105), *ITGA6* (CD49f) and *TFRC* (CD71) were close to the threshold of significance for upregulation (log2FC = 1.74 (*ENG*), 1.77 (*ITGA6*) 1.84 (*TFRC*) (Supplementary Figure S3). Furthermore, *MATN3* was upregulated in chondrocytes as well as *VCAM1* (*CD106*) in osteocytes. In contrast, we found a downregulation of *SOX11* and *MME* in osteocytes and *CD70* and *NT5E* in adipocytes.

### 3.4 Exon usage alterations in differentiating BM-MSC

Differential exon usage analysis was performed for the same contrasts as tested for DEGs: We called differences between BM-MSCs and PBMCs, as well as differences between BM-MSC and adipocytes, chondrocytes, or osteocytes. In total, 2,93,078 exons were aligned and counted. Among these exons, 20,507 (related to 7,126 genes) with padj < 0.05 differed in terms of exon usage in either PBMC or BM-MSCs, and 7,148 exons (related to 3,820 genes) with |l2FC|>2 were significantly differentially expressed (Supplementary Table S10).

**FIGURE 7:**
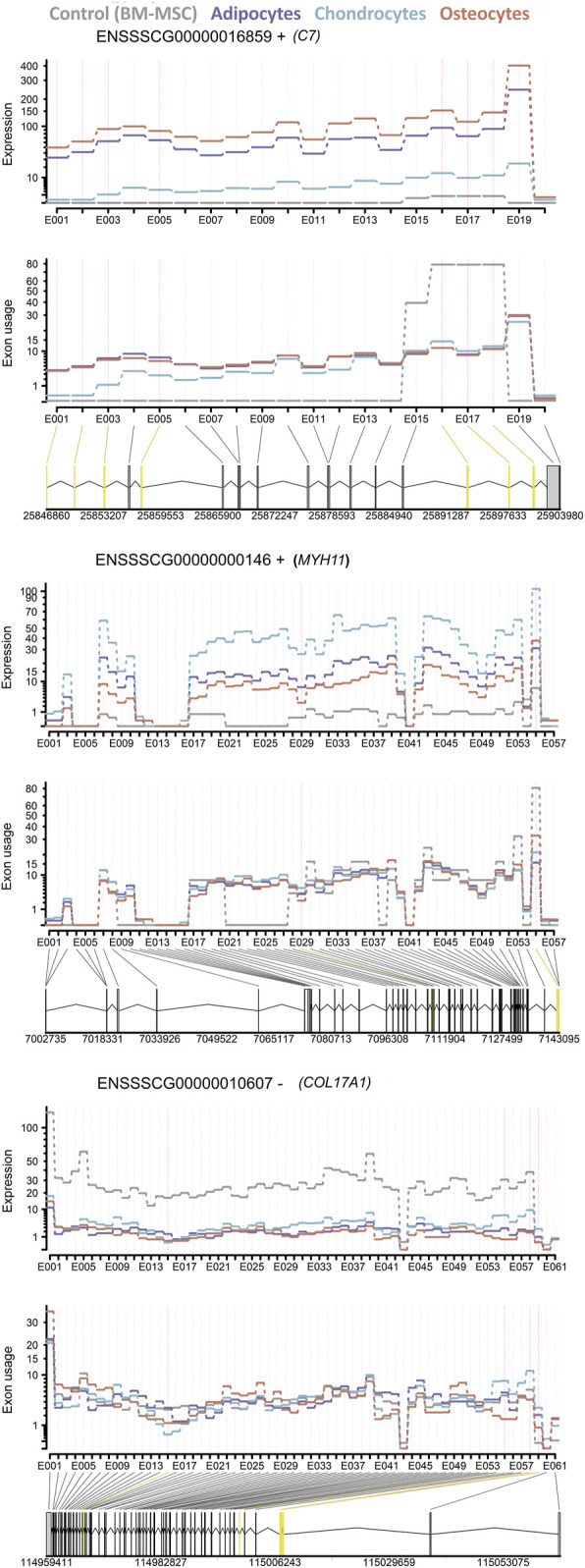
Differential exon usage of three DEGs *C7*, *MYH11* and *COL17A1*. The expression level and exon usage of each exon of (top) *C7*, (middle) *MYH11* and (bottom) *COL17A1* for all cell lineages are displayed. The differential exon usage for each cell lineage was tested against BM-MSC as control. Common significant differentially used exons in all three cell lineages are highlighted in yellow.

Furthermore, exon usage analysis among adipocytes, chondrocytes, osteocytes and BM-MSCs (as control) revealed for all four groups 2,860 exons (related to 7,612 genes) with potential significant effects (padj < 0.05) on the phenotypes (Supplementary Table S11). Additionally, the exon expression was compared between each cell type to the control, resulting in 356 exons (related to 283 genes) in adipocytes, 3,346 exons (related to 2,177 genes) in chondrocytes and 2.735 exons (related to 1,810 genes) in osteocytes within the significance threshold |l2FC|>2 for differential expression of exons. Additionally, we found the three genes *C7*, *COL17A1* and *MYH11*, called as part of the group of the common 18 DEGs, in differentiated cell lineages to contain significantly differential exon usage in six (*C7*) or one (*COL17A1* and *MYH11*) exons, respectively (Figure 7).

### 3.5 WGCNA highlights clusters of co-expressed genes associated with BM-MSC lineage

**FIGURE 8:**
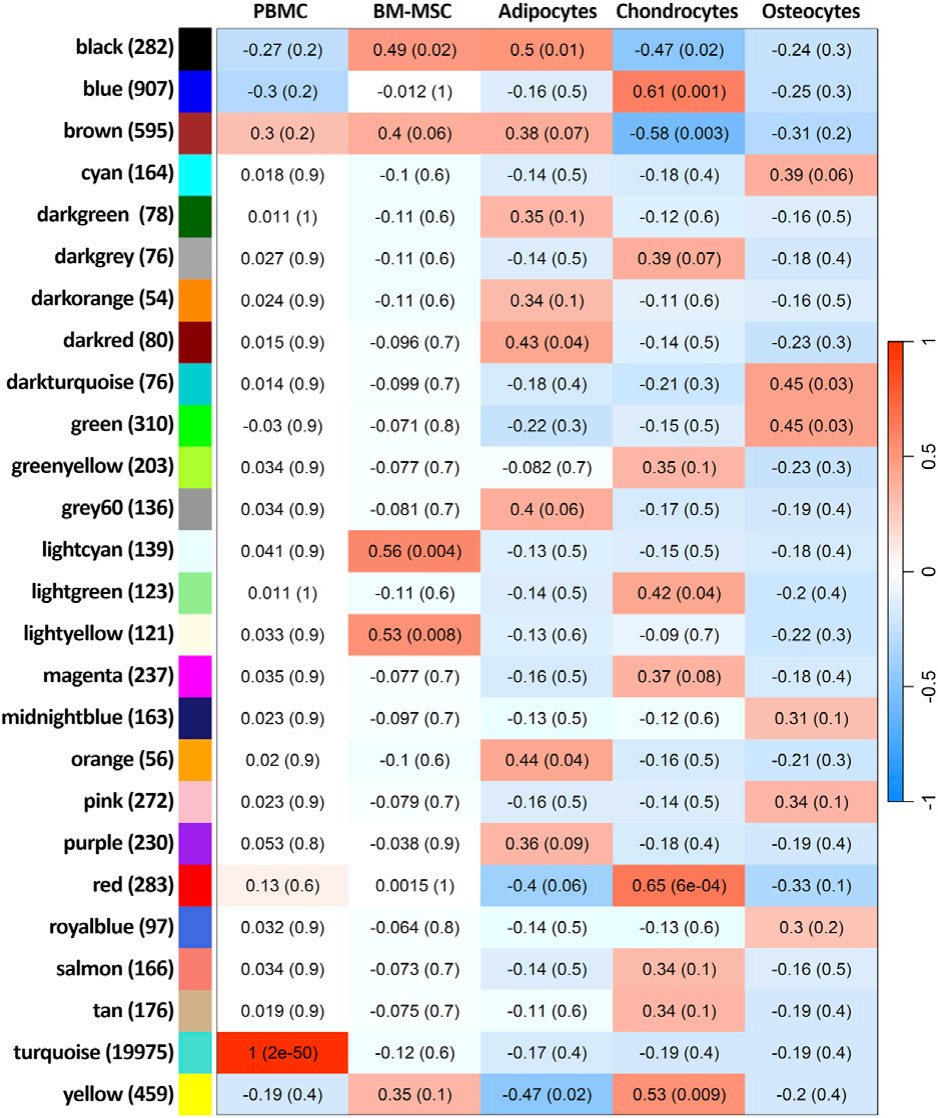
Weighted gene co-expression network analysis. The heat map presents the correlation between modules and cell types. The size of each module is mentioned in parenthesis next to the module’s name. FDR of each correlation is present in parenthesis on its corresponding heat-cell.

The complete transcriptome of 25,484 expressed genes among all five groups of cells was fitted into 26 weighted co-expressed gene networks (Figure 8; Supplementary Table S12). Only one module was exclusively correlated to PBMCs (correlation = 1 and *p-*value < 2 × 10^−50^), representing the largest module in terms of number of genes (19,976 genes, module “turquoise”). This module did not correlate with BM-MSC and its lineages. Therefore, the co-expressed genes within this module were specific to PBMC transcriptome. Furthermore, among the 26 clusters, the smallest module contained 55 co-expressed genes and held no significant correlation with any phenotype (module “darkorange”). The module with the highest correlation (correlation = 0.56 and *p-*value < 0.04) to BM-MSC phenotype was “lightcyan” with 140 co-expressed genes. However, no DEG related to BM-MSC could be identified within this module. The majority of DEGs in BM-MSC (versus PBMCs) were detected in module “turquoise” (6,162 out of 6,285 DEGs) and “yellow” (61 out of 6,285 DEGs). Adipocytes were correlated significantly with four different modules (“black,” “darkred,” “orange” and “yellow”). Chondrocytes showed a strong significant correlation with six different modules (“black” “blue” “brown” “lightgreen” “red” and “yellow”) and osteocytes were correlated significantly with only two modules, “darkturquoise” and “green”. In addition, all the significant clusters were investigated using enrichment analysis (Supplementary Table S13).

### 3.6 Fluorescence microscopy investigation of cell cycle stages as well as genes from “regulation of actin cytoskeleton” pathway on protein level

**FIGURE 9:**
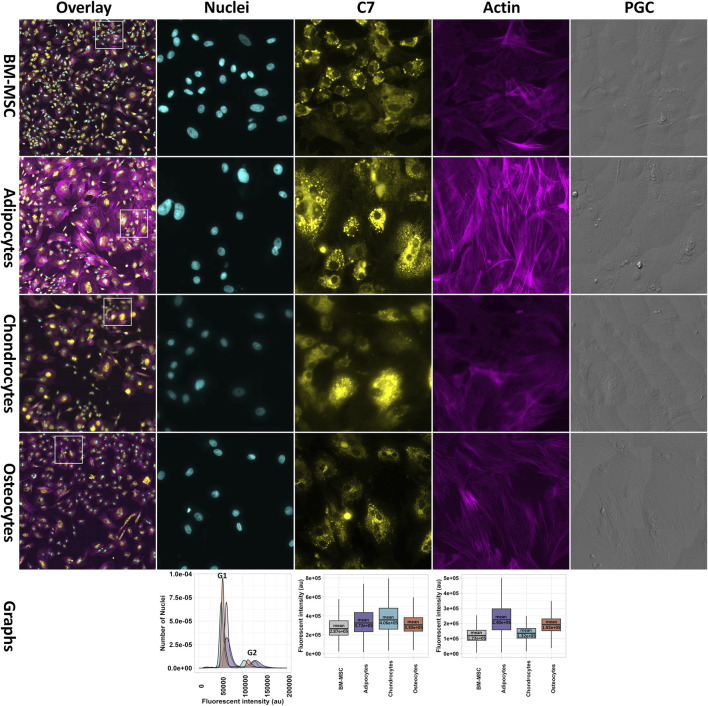
Fluorescence microscopy images of fixed BM-MSC and its differentiated cell lineages. The fixed cells were stained for nuclei (Hoechst, blue), C7 (anti-C7; yellow) and actin (Phalloidin, purple). The bottom panel, from left to right, shows the analytical graphs for fluorescent intensities for DNA content (left), anti-C7 (middle) and anti-actin (right). The histogram at the left shows the DNA content with fluorescent intensity on the *x* axis. The more intense signal is the indicator of G2 cycle due to DNA duplication. The plots display mean fluorescent intensity of C7 (middle) and actin (right). The plots show significant increase in C7 and actin in the differentiated cells in comparison to BM-MSC. The mean value of each intensity is written in each corresponding plot. All testes are significant with FDR <0.05 (the data is not shown).

Active proliferation of all tested cell types was shown by DNA counter stain. The fluorescent intensity signals from DNA content in BM-MSC and differentiated lineages represented cell cycle stages G1 and G2 as indicators of actively mitotic cells. In our results, we found that osteocytes showed more cells in G1 cycle, whereas adipocytes had less cells in G1 stage. However, no significant changes were be observed in G2 stage among all cells (Figure 9).

Furthermore, fluorescent microscopy approach was used for validation of RNA-seq results on protein level regarding the “regulation of the actin cytoskeleton” in BM-MSC and differentiated cells. We targeted not only C7 as an upstream protein in the pathway but also actin filaments as the final product of the pathway (please refer to Figure 5). The fluorescent intensities of both C7 and actin in differentiated cells (adipocytes, chondrocytes, and osteocytes) showed a significant increase in comparison to BM-MSC (Figure 9). Alongside the detected fluorescent signal, the images displayed visually different patterns and rearrangements of the actin filaments, which indicated a more active pathway in the differentiated cells.

## 4 Discussion

In our work, we studied comprehensive transcriptome profiles linking BM-MSC gene expression patterns to cell-specific characteristics and highlighting transcriptome dynamics during targeted differentiation. To our knowledge, this is the first study in pigs, which investigates a profound list of genes coding for CD markers in BM-MSC to highlight expression patterns of these key stem cell surface markers as potential candidates for future improved cell-type characterization.

Our transcriptome data shed light to the debate regarding the expression of well-known markers such as CD73 (*NT5E*) and CD105 (*ENG*) in porcine BM-MSC. We observed the gene encoding the surface marker CD105 to be below the significance threshold but with an absolute log2 fold change approximating |log2FC|)>2 (log2FC = 1.74 and padj = 1.9 × 10^−6^). This finding that CD105 is expressed in BM-MSC was also confirmed with our image analysis in which we used anti-CD105 anti-pig antibody. We assume, that this finding might explain the divergent results in previous studies, reporting on either no expression of CD105 or a “mild positivity” as suggested in flow cytometry data (Juhásova et al., 2011; Schweizer et al., 2020). Similarly, we found the gene encoding CD73 to be strongly upregulated in porcine BM-MSC, contradicting a previous study reporting on its absence (Schweizer et al., 2020). With regard to these discrepancies, we follow Prinz’ (Prinz, 2017) reasoning, who suspects the low number of commercially available porcine antibodies and subsequent potential use of alternatives from human or mice to be the cause for differential results in different studies. Consequently, our list of expression patterns of genes encoding cell surface markers might be of help for future improved porcine stem cell characterization.

Furthermore, by using PBMCs as benchmark for our study, we found genes pointing to the immunomodulatory properties of BM-MSCs. We identified clusters of more than 800 genes involved in the regulation of the immune system. As demonstrated in previous studies (Khatri et al., 2015; Liu et al., 2012; Uccelli et al., 2007; Blanco et al., 2016), several of these genes belong to surface markers (such as CD4, CD8, CD19 and CD80), interleukin families and their receptors (IL1R, IL2R, IL4, IL10 and IL12 to name a few) and different growth factors (such as FGF2, FGF7 and FGF10) as well as interferons (IFNs) and tumor necrosis factors (TNFs). These results are in agreement with the findings of functional studies of MSCs, highlighting the inhibitory effect of BM-MSCs on the proliferation of T cells, B cells, dendritic cells and natural killer cells (Uccelli et al., 2007; Russell et al., 2016). It was suggested that this ability of MSCs could even be used to dampen immune-mediated diseases and transplant rejection (Uccelli et al., 2007).

In addition to these findings, we identified very interesting expression patterns in BM-MSC, which are obviously characteristic for this cell type with regard to its stemness properties; According to our analysis of DEGs for BM-MSC, we found a significant gene enrichment for “Rap1 signaling pathway” (KEGG pathway ssc04015) suggesting its activation, as well as interactions with “Extracellular matrix receptors” (KEGG pathway ssc04512). Interestingly, the components of Rap1 pathway have been shown to regulate paracrine MSC activities as well as promote cell survival by activating DNA double-strand break repair mechanisms (Ding et al., 2018; Khattar et al., 2019). In addition, Rap1/PI3K/Akt axis of this pathway was found to be involved in cell proliferation, migration and differentiation (Chen et al., 2022; Takahashi et al., 2013; Jiang et al., 2024; Jiang et al., 2019). Thus, these findings underline the differentiation capacity of BM-MSC (Chen et al., 2022).

This nature of BM-MSC was likewise highlighted by our findings of 18 common DEGs in all differentiated cell lineages. Collectively, these DEGs were apparently associated with either characteristic common molecular processes underlying the core stem cell properties of self-renewal or the generation of differentiated progeny, as referred to stemness (Douglas et al., 2014). Among these genes, we identified *FGF19*, downregulated in all three datasets, which is known to promote epithelial-mesenchymal transition (EMT) and self-renewal capacity of cancer stem cells, and to induce cell cycle arrest in differentiated chondrocytes (Wang et al., 2021; Zhao et al., 2016; Chen et al., 2023). Furthermore, our data also revealed a downregulation of *IGFBP6*, which is supposed to result in the inhibition of EMT and activation of differentiation (Cui et al., 2011; Nikulin et al., 2018) as well as *EGR1*, meditating actin assembly and mechanotransduction signaling in stem cells in response to cytoskeletal tension (Bleher et al., 2020; Baek et al., 2022). *PTH1R* is another example, which was upregulated in our datasets and subsequently might be involved in the initiation of bone formation and differentiation through activation of parathyroid hormone and Wnt signaling pathway (Yu et al., 2012). Furthermore, *SMOC2*, which was upregulated in all data-sets, could probably act as an enhancer in activating PI3K-Akt signaling pathway and subsequently promote differentiation as previously suggested (He et al., 2023). These findings suggest that these common DEGs might be key players represented as “BM-MSC differentiation driver markers.

Among these potential differentiation driver genes, ten DEGs were also found to be differential in BM-MSC vs. PBMC. This strongly suggests that these genes might be of importance for the maintenance of the BM-MSC phenotype. For two out of the remaining eight genes, *C7* and *MYH11*, we found significant differential exon usage in addition to their upregulation in all differentiated cell-types. This finding follows previous assumption that the encoded proteins of these genes might play a significant role in the activation of stress fibers in the actin cytoskeleton (KEGG pathway ssc04810 (Kanehisa and Goto, 2000; Kanehisa, 2019; Kanehisa et al., 2023)) and therefore control cell proliferation, migration and lineage commitment (Muller et al., 2013; Burnette et al., 2011; Clarke and Martin, 2021). Notably, this pathway meditates PI3K signaling through growth factors such as *FGF19* (KEGG pathway ssc04810) as well as MAPK signaling pathway promoting the mechanically induced signal transduction and differentiation (Muller et al., 2013).

In summary, we presume that for porcine BM-MSC, Rap1 signaling and subsequently its downstream pathways PI3K-Akt as well as MAPK signaling are essential players for the cellular functionality and maintenance of its stemness. The increase in PI3K-Akt activity might lead the cell toward proliferation and differentiation. In addition, we assume that changes in the regulation of actin cytoskeleton during differentiation, might not only result in cell morphological changes but also facilitate the activity of PI3K and MAPK cascades. As potential key players in this differentiation process, our data highlight 18 candidate “BM-MSC differentiation driver markers.” Subsequently, this study offers a comprehensive molecular phenotype of porcine BM-MSC and elucidates its potential underlying mechanisms *in vitro*.

## Data Availability

The datasets presented in this study can be found in online repositories. The names of the repository/repositories and accession number(s) can be found in the article/Supplementary Material.

## Supplementary material

SUPPLEMENTARY FIGURE S1 Heatmap of sample-sample distance and clustering. The heatmap visualizes the results of similarity and hierarchical clustering between each cell type and its replicates based on Euclidian distances. Distances are displayed on each cell of the heatmap.

SUPPLEMENTARY FIGURE S2 PCA plots from differential gene expression analysis. The PCA plot shows the variances among the BM-MSC, adipocytes, chondrocytes, and osteocytes datasets.

SUPPLEMENTARY FIGURE S3 Expression of not differentially expressed BM-MSC markers. The bar chart display the log2FC of known but not differentially expressed MSC markers in porcine BM-MSC, adipocytes, chondrocytes and osteocytes. Padj of each gens is displayed on its respective bar.
