# Supplementary figures and images for: Deciphering transcriptome patterns in porcine mesenchymal stem cells promoting phenotypic maintenance and differentiation by key driver genes
Source: Front Cell Dev Biol. 2024 Nov 6;12:1478757. doi: 10.3389/fcell.2024.1478757 (PMC11576426; doi:10.3389/fcell.2024.1478757)

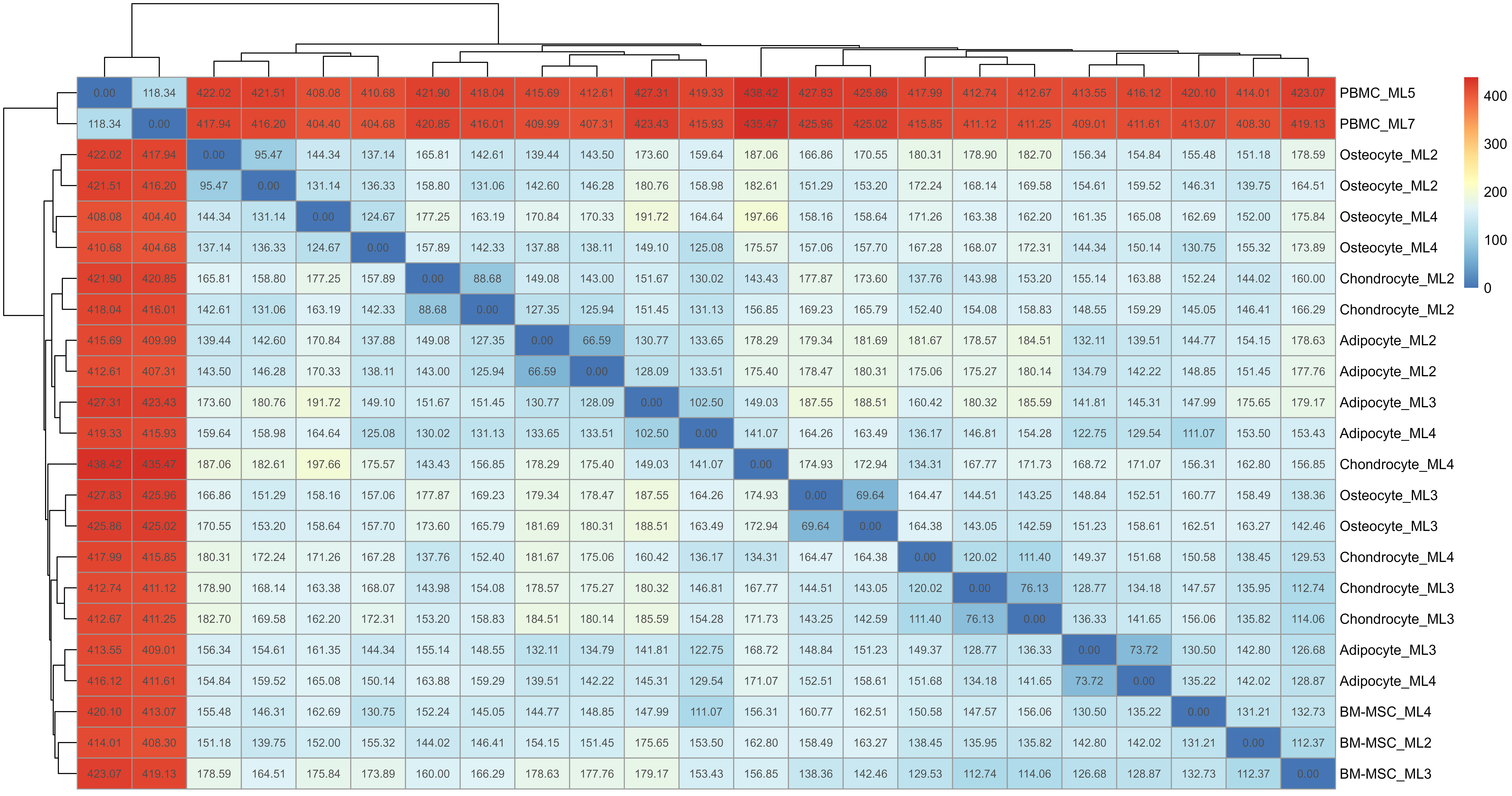

Supplement: Supplementary file 5 [file Image1.jpeg]

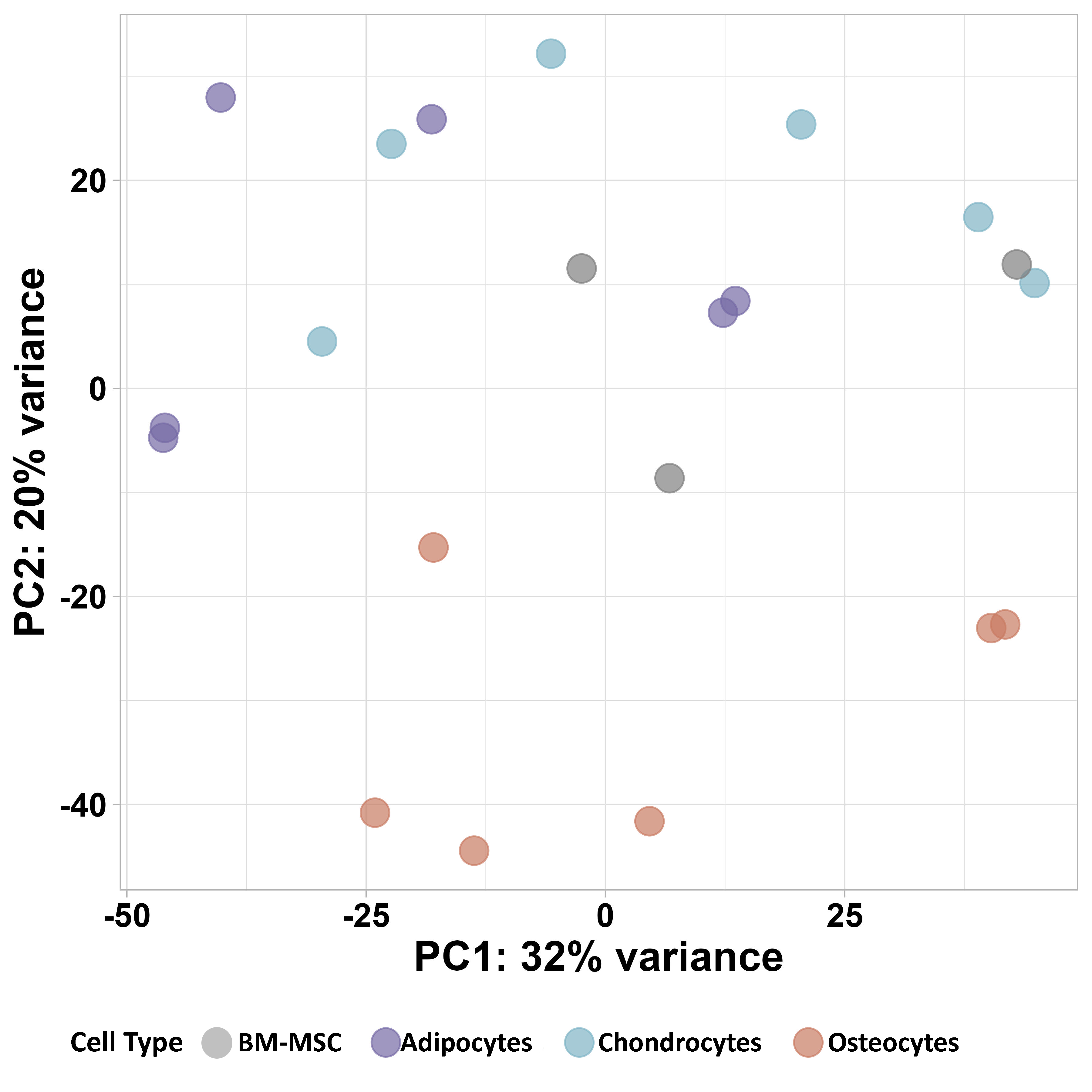

Supplement: Supplementary file 6 [file Image2.jpeg]
